# The State and the Mosquito

**Published:** 1904-03

**Authors:** 


### Abstracts and Selections.

### The State and the Mosquito.

At last the mosquito question is regarded as of national importance, and men of light and learning in all branches of life have been brought to believe and to appreciate the fact that the mosquito is a foeman by no means to be despised. The mosquito has long been regarded as an excellent medium for jesting, and the evils which it brings in its wake have not been recognized at their full significance. Of course, up to a comparatively recent date, the disease-bearing properties of certain mosquitoes were not known; but even now, the humorous side of the matter is more apt to crop up than is the grave. The convention called by Governor Murphy, of New Jersey, and which held its session a short time ago in New York, discussed in all its bearings the situation as regards the mosquito. The meeting was attended not only by men of science, but by those prominent in various other ways. The suggestions advanced with a view of exterminating or of keeping out the insects were many, some of which seemed feasible, and some chimerical. Mr. Walter C. Kerr, of Staten Island, was of the opinion that if the city of New York would begin the work of extermination by spending $2000, the rural communities would continue it until mosquitoes ceased to exist. Dr. L. O. Howard sent an interesting letter, and many others spoke or wrote. Finally resolutions were adopted recommending that a provisional committee be appointed by the convention to consider the formation of a central organization of national character, with which local bodies might co-operate in conducting a gigantic mosquito crusade.

The most gratifying and encouraging feature of the convention is that it signifies the awakening of the American people to the truth, that the mosquito has wrought and is capable of wreaking, an immense amount of injury to life and property. These insects have done incalculable harm in all parts of the world. Several of the European nations, and Japan in the far East, have already organized vigorous crusades against the mosquito. This country has been slow in acting, but as soon as it is thoroughly well understood how great is the power for harm possessed by the mosquito, the United States will take measures which will equal or surpass those of other lands in suppressing the pest.—*Medical Record*, January 2d.

